# Intermittent short-term low dose cyclosporine may help break the itch-scratch cycle in uremic pruritus: A retrospective study

**DOI:** 10.1016/j.jdin.2026.04.017

**Published:** 2026-05-04

**Authors:** Hyoung Soo Park, Heung Soo Kim, Hee Young Kang, Jin Cheol Kim

**Affiliations:** a Department of Dermatology, Ajou University of Medicine, Suwon, South Korea; b Department of Nephrology, Ajou University of Medicine, Suwon, South Korea

**Keywords:** cyclosporine, epidemiology, itch, pruritus, renal

*To the Editor:* Uremic pruritus is a common cause of chronic itch, and like other forms of chronic itch, it is crucial to break the itch-scratch vicious cycle as early as possible to prevent exacerbation.^1^^,^^2^ Cyclosporine, a selective calcineurin inhibitor, has been used for various dermatologic diseases, including refractory chronic itch because of its rapid onset.^3^ However, cyclosporine use in uremic pruritus is limited due to concerns regarding renal toxicity. The side effects are known to be dose dependent and related to the duration of therapy. Although it is reported that renal toxicity from low dose (<5 mg/kg/day) cyclosporine is rare,^4^^,^^5^ data on optimal dosing regimens that balance efficacy with renal safety are limited (ref). Therefore, this retrospective study investigated the real-world efficacy and safety of intermittent short-term (2-12 weeks) low dose cyclosporine therapy for breaking itch-scratch cycle in uremic pruritus.

Sixteen uremic pruritus patients having received oral cyclosporine corresponding to 11 males and 5 females, with a mean age 69.9 years. Most patients had diabetes related to CKD (Table I). The itch did not improve with any treatments including antihistamines, methotrexate, and phototherapy. We initiated microemulsion-cyclosporine with mean daily dose of 1.4 mg/kg/d for 2 weeks after the patient’s consent. The initial 2-week period of fixed daily dosing was defined as “rescue therapy,” intended to rapidly break the itch-scratch cycle. Following the rescue therapy, patients entered the “intermittent therapy” phase (up to a maximum of 12 weeks). During this phase, treatment consisted of gradual dose tapering or pro re nata (PRN; as-needed) administration. For the PRN protocol, patients were instructed to resume medication only upon the recurrence of pruritus and to discontinue use once symptom relief was achieved. Throughout these treatment periods, all participants received regular consultations not only from the dermatologist (J.C.K., H.Y.K.) but also from the nephrologist (H.S.K.) to check nephrotoxicity.

**Table I:** Clinical characteristics in 16 patients with uremic pruritus treated with cyclosporine

Patients no.	Sex	Age (y)	Total treatment duration (wk)∗	Initial dose		Initial serum creatinine (mg/dL)	Comorbidities	Past medication	VAS for pruritus		
				(mg/d)	(mg/kg/d)				Initial	After rescue therapy	End of administration
1	F	88	8	100	1.6	1.0	HTN, DM	AH	7	6	4
2	M	82	4	75	0.9	3.5	HTN, gout	AH, steroid	6	3	2
3	F	69	8	100	1.3	1.7	-	AH, steroid	7	5	2
4	F	73	4	100	1.6	1.6	-	AH, steroid	7	4	2
5	M	89	8	100	2.0	1.4	HTN, DM	AH	8	6	4
6	M	70	8	100	1.8	2.6	HTN, DM	AH, phototherapy	5	4	3
7	M	76	8	75	1.2	1.5	BPH, dyslipidemia, DM, DM retinopathy, cataract	AH, phototherapy	6	3	3
8	M	54	2	25	1.5	1.7	HTN, immunoglobulin A nephropathy	AH, steroid, phototherapy	6	4	4
9	M	39	12	200	3.5	7.5	HTN, dyslipidemia	AH, steroid	7	5	3
10	F	76	8	100	1.3	1.8	HF, DM, CAOD	AH	5	3	2
11	M	81	12	75	1.5	1.3	-	AH, steroid	6	4	3
12	M	52	12	200	3.2	1.3	-	AH, steroid	7	6	4
13	F	84	12	100	1.8	1.4	-	AH, steroid	7	7	5
14	M	47	4	75	1.6	3.3	HTN, DM	AH, steroid	6	3	2
15	M	69	8	75	1.4	1.7	Hepatitis B, CAOD, Parkinson’s disease	AH, steroid	7	5	3
16	M	69	12	75	1.3	1.5	DM	AH	6	5	4

*AH*, Antihistamine; *BPH*, benign prostate hyperplasia; *CAOD*, coronary artery obstructive disease; *DM*, diabetes mellitus; *F*, female; *HF*, heart failure; *HTN*, hypertension; *M*, male; *VAS*, visual analogue scale.

∗ Patients commonly received a 2-week rescue therapy, followed by a gradual dose tapering or pro re nata (PRN) administration of cyclosporine until relief of itch or maximum of 12 weeks.

Treatment efficacy was evaluated by change of visual analogue scale (VAS) for pruritus. Safety was evaluated by monitoring serum creatinine levels at standardized intervals (baseline, 2, 4, 8, and 12 weeks) and reviewing other systematic symptoms. The VAS for pruritus after last cyclosporine administration (mean of 8.5 weeks) significantly decreased compared with baseline, but serum creatinine levels did not significantly change during treatment (Fig 1). Mean initial serum creatinine level was 2.2 mg/dL and most of the patients (15 out of 16 patients) showed stable level of serum creatinine without abrupt or unexpected increase and other systematic symptoms during the treatment. Only 1 patient (patient 8) ceased cyclosporine due to abrupt serum creatinine elevation with uremic symptoms, which normalized afterward (Supplementary Fig 1, available via Mendeley at https://data.mendeley.com/datasets/rx6cjytmcz/1).

**Fig 1:**
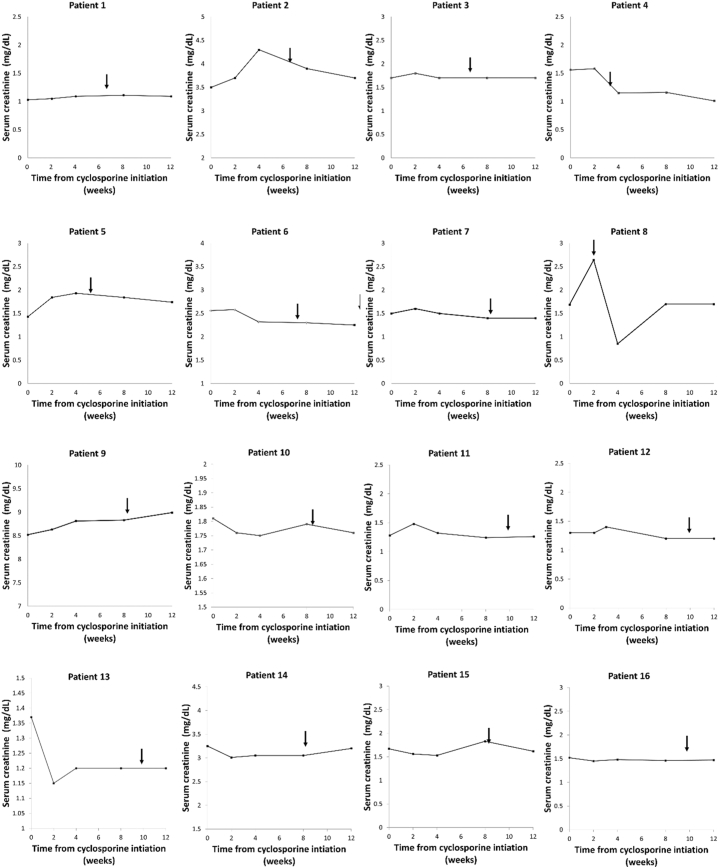
Changes in serum creatinine levels in each patient with uremic pruritus during cyclosporine administration. Each graph represents the time course of serum creatinine levels for an individual patient (Patients 1-16) during intermittent short-term (2-12 wks) low-dose cyclosporine therapy. The arrow indicates the specific time point of the last cyclosporine administration for each patient. Note that most patients maintained stable creatinine levels, while Patient 8 exhibited a transient elevation that normalized after discontinuation.

This study shows that intermittent short-term low dose cyclosporine can adequately control refractory uremic pruritus. Most patients achieved rapid relief during the 2 weeks of rescue therapy and maintained or improved condition throughout the course of intermittent therapy. Taking into account the findings from our results, having CKD alone does not automatically render the use of cyclosporine for pruritus treatment as an absolute contraindication. Therefore, intermittent short-term cyclosporine use may be considered as a second-line therapeutic option for refractory uremic pruritus with careful monitoring of serum creatinine levels and other side effects, and further long-term prospective and large-scaled study is needed to validate efficacy, safety, and post-cessation sustainability.

## Conflicts of Interest

None disclosed.
